# Prevalence of burnout among Brazilian Army soldiers during the COVID-19 pandemic: a cross-sectional analysis

**DOI:** 10.47626/1679-4435-2025-1467

**Published:** 2025-12-29

**Authors:** Rômulo de Oliveira Fraga, Alessandra S. A. Fraga, Angelica Alessandra Maciel Conte, Jovito Adiel Skupien, Natielen Jacques Schuch

**Affiliations:** 1 Franciscan University (UFN), Santa Maria, RS, Brazil.

**Keywords:** occupational stress, burnout syndrome, armed forces health, COVID-19, estresse ocupacional, esgotamento psicológico, militares, covid-19

## Abstract

**Introduction:**

The COVID-19 pandemic significantly impacted the mental health of frontline workers, including military personnel.

**Objectives:**

To determine the prevalence of burnout syndrome among Brazilian Army personnel during the pandemic and identify associated predictive variables.

**Methods:**

A cross-sectional study was conducted with 602 volunteer military personnel in the city of Santa Maria, Brazil. The Maslach Burnout Inventory was used to assess burnout syndrome, defined by high levels of emotional exhaustion, depersonalization, or low personal accomplishment. Logistic regression was performed to identify associated factors.

**Results:**

The prevalence ofburnout syndrome was 48.7%. High levels of emotional exhaustion, depersonalization, and low personal accomplishment were found in 53.8%, 58.4%, and 27.0% of participants, respectively. Emotional exhaustion was more common in those with over 10 years of service (p = 0.001), higher rank (p < 0.001), and age > 40 years (p = 0.008). Low personal accomplishment was associated with lower rank (p = 0.007) and administrative duties (p = 0.032).

**Conclusions:**

Burnout syndrome was highly prevalent among military personnel in Brazil during the pandemic, with findings similar to those observed in other professional groups.

## Abstract (pt)

**Introdução:**

A pandemia de covid-19 impactou significativamente a saúde mental dos trabalhadores da linha de frente, incluindo os militares.

**Objetivos:**

Determinar a prevalência da síndrome de *burnout* entre militares do Exército Brasileiro durante a pandemia e identificar variáveis preditoras.

**Métodos:**

Estudo transversal realizado com 602 militares voluntários em Santa Maria, Brasil. Utilizou-se o Maslach Burnout Inventory para avaliar a síndrome de *burnout*, definida por altos níveis de exaustão emocional, despersonalização ou baixa realização pessoal. A regressão logística foi aplicada para identificar fatores associados.

**Resultados:**

A prevalência de síndrome de *burnout* foi de 48,7%. Exaustão emocional, despersonalização e baixa realização pessoal foram observadas em 53,8%, 58,4% e 27,0% dos participantes, respectivamente. A exaustão emocional foi mais comum entre os militares com mais de 10 anos de serviço (p = 0,001), de patente mais alta (p < 0,001) e idade superior a 40 anos (p = 0,008). A baixa realização pessoal esteve associada à patente mais baixa (p = 0,007) e à atuação em funções administrativas (p = 0,032).

**Conclusões:**

A síndrome de *burnout* apresentou alta prevalência entre militares durante a pandemia, em níveis comparáveis aos encontrados em outros grupos profissionais.

## INTRODUCTION

The Brazilian Army is a military force responsible for land-based operations and is a permanent and regular national institution established in the 1988 Constitution of the Federative Republic of Brazil. Its organization is grounded in hierarchy and discipline, and its mission includes the defense of the Fatherland, the safeguarding of constitutional powers, and, when requested by any of these powers, the maintenance of law and order.^1^ During the COVID-19 pandemic, the Army participated in Operation Covid-19, conducting disinfection of public facilities, providing logistical support for the transfer of equipment and basic food baskets, assisting in the transportation and burial of victims, producing essential supplies in a military pharmaceutical laboratory, setting up health-screening posts to support communities, and mobilizing blood-donation campaigns to mitigate shortages, among other activities.

The military profession is characterized by constant emotional pressure, demands for continuous preparedness, recurrent emergency situations, exhausting work schedules, and intense interpersonal relationships. These conditions, among others, may contribute to the development of mental and behavioral disorders, including burnout syndrome (BS).^2^

### CORRELATION BETWEEN BURNOUT SYNDROME AND ANXIETY IN MILITARY PERSONNEL

Essential workers operating in highly challenging contexts are expected to make complex decisions and frequently express concerns about their risk of exposure and that of their families. Such circumstances can negatively impact mental health in both the short and long term.^3^

The collapse of the health system caused by the emergence of the COVID-19 pandemic, an infectious disease caused by the novel coronavirus SARS-CoV-2 (2019-nCoV), affected all sectors of the Brazilian population, particularly frontline workers such as health care professionals and security personnel. The Armed Forces were incorporated into this broader context of pandemic-related stress and were required to intensify their activities due to increased operational demands, experiencing significant changes in their usual duties. Consequently, military personnel faced circumstances with substantial implications for their mental health, given the greater risk of stress linked to prolonged exposure to the virus and the need to adhere to crisis-management policies aimed at protecting citizens.^4^

Brazil reported the second highest number of COVID-19-related deaths worldwide, following the United States. To the best of our knowledge, this is the first study to examine BS among members of the Brazilian Army during the COVID-19 outbreak.^5^ The main objective of this study was to investigate the prevalence of BS in the context of the COVID-19 pandemic in Brazil and to identify predictive variables associated with burnout levels among these professionals.

## METHODS

This study followed the reporting standards for retrospective research in accordance with the recommendations for Strengthening the Reporting of Observational Studies in Epidemiology (STROBE).^6^

### DESIGN

This cross-sectional study was conducted between March 9 and August 14, 2020, involving volunteer military personnel from Brazilian Army units based in the city of Santa Maria, Rio Grande do Sul, Brazil.

### ETHICAL ASPECTS

The study complied with all guidelines and regulations governing research involving human participants, as established by Resolution No. 422 of December 22, 2012, of the Brazilian National Health Council. All participants signed an Informed Consent Form (ICF). Anonymity and confidentiality were strictly preserved. The study received approval from the Medical Ethics Committee of the Hospital Geral de Santa Maria and authorization from the Chief of the General Personnel Department of the Brazilian Army.

### PARTICIPANTS

Eligible participants included voluntary Brazilian Army personnel serving in the city during the data collection period. Exclusion criteria included individuals on leave at the time of data collection, those with incomplete biochemical examination reports, and those with incorrectly completed questionnaires.

### VARIABLES

Sociodemographic and military-service variables were collected, including age, sex, and career-related information. BS was assessed using the Maslach Burnout Inventory (MBI), the most widely used instrument for evaluating burnout, validated for use in Brazil in 1995.^7^ MBI scores were interpreted according to the cutoff points recommended by the scale developers.^8^ Consistent with previous studies,^9^,^10^ burnout was defined by the presence of high levels of emotional exhaustion, depersonalization, or low personal accomplishment.^9^ Anthropometric measurements were obtained after participants removed their shoes and heavy outer garments. Body weight (kg) was measured to the nearest 0.1 kg using a balance-beam scale, and height (cm) was measured using a stadiometer. Body mass index (kg/m^2^) was calculated, and nutritional status was classified according to World Health Organization criteria.^11^

Body composition was evaluated using anthropometry and bioelectrical impedance analysis (BIA). BIA was performed using a tetrapolar single-frequency apparatus (50 kHz and 0.8 mA; Biodynamic-450, Biodynamics Corporation, USA) applied to the skin using adhesive electrodes. Phase angle (PhA), derived from BIA, was determined as previously and its values were calculated using the formula: PhA = arctangent (reactance/resistance) x (180°/n). Body cell mass and fat mass were recorded according to device parameters. All measurements were performed after a 12-hour overnight fast, with participants having emptied their bladder and remaining in the supine position for 15 minutes prior to assessment.^12^

### STATISTICAL ANALYSIS

Data were analyzed using the Statistical Package for Social Sciences (SPSS), version 21.0. The normality of quantitative variables was assessed using the Kolmogorov-Smirnov test. Results were described using measures of central tendency (mean and median) and dispersion (SD and interquartile range), depending on the symmetry of the distribution. Comparisons between quantitative variables were conducted using Student’s t-test, Mann-Whitney U test, and ANOVA with Bonferroni post-hoc adjustment. Associations between categorical variables were examined using Pearson’s chi-square test and Fisher’s exact test. Multivariate analysis was performed using a binary logistic regression model. Statistical significance was set at p < 0.05.

## RESULTS

A total of 602 military personnel (N = 602) were evaluated, with a mean age of 34.2±9.3 years. Most of the sample was composed of men, representing 72.8% of participants. Regarding age distribution, 35.4% were between 21 and 30 years old, 37.5% were between 31 and 40 years old, and 27.1% were older than 40 years. In addition, 60.0% had less than 10 years of service, while 40.0% had served for 10 years or more. Operational duties were performed by 54.2% of the sample, while 45.7% carried out administrative (bureaucratic) activities.

Assessment of nutritional status revealed that most professionals were classified as overweight or obese (62.5%). With respect to body fat percentage, 13.0% demonstrated values within the average range, 33.2% showed better-than-average values, and 53.8% presented worse-than-average values. The mean scores for the MBI subscales were 16.1± 10.6 for emotional exhaustion, 5.7±4.9 for depersonalization, and 36.8±7.5 for low personal accomplishment. Based on these results, the overall prevalence of BS was 48.7% (Table 1).

**Table 1:** General characteristics of the participants (n = 602)

Variable	Value
Gender	
Male	438 (72.8)
Female	164 (27.2)
Age (years)	
21-30	213 (35.4)
31-40	226 (37.5)
> 40	163 (27.1)
Rank	
Superior Officers	25 (4.1)
Captains and Lieutenants	170 (28.2)
Warrant Officers and Sergeants	317 (57.7)
Corporals and Privates	90 (15.0)
Length of service (years)	
< 10	361 (60.0)
> 10	241 (40.0)
Work category	
Operational	326 (54.2)
Bureaucratic	276 (45.8)
Nutritional status	
Eutrophic	226 (37.5)
Overweight/obesity	376 (62.5)
Classification of body fat (%)	
Better than average	200 (33.2)
On average	78 (13.0)
Worse than average	324 (53.8)
Age, mean ± SD	34.2±9.3
Maslach Burnout Inventory Score, mean ± SD	
Emotional exhaustion	16.1±10.6
Depersonalization	5.7±4.9
Low personal accomplishment	36.8±7.5
Classification of burnout	
No	309 (51.3)
Yes	293 (48.7)
Classification of MetS	
No	446 (74.1)
Yes	156 (25.9)
MetS components	
Blood pressure	
Systolic mmHg, mean ± SD	124.0±13.6
Diastolic mmHg, mean ± SD	80.6±11.3
Normal	317 (52.7)
High	285 (47.3)
Waist circumference (cm), mean ± SD	85.4±10.3
Normal	375 (62.3)
Increased	227 (37.7)
Glucose (mg/dL), mean ± SD	90.6±12.7
Normal	477 (79.2)
High	125 (20.8)
Triglycerides (mg/dL), mean (IQR)	97 (71-142)
Normal	469 (77.9)
High	133 (22.1)
HDL-c (mg/dL), mean ± SD	51.7±16.5
Normal	439 (72.9)
Low	163 (27.1)

Data are presented as n (%), unless otherwise specified. HDL-c = high-density lipoprotein cholesterol; IR = interquartile range; MetS = metabolic syndrome.

It was observed that professionals performing bureaucratic duties were 56.5% more likely to present BS (OR 1.565; 95%CI 1.134-2.161; p = 0.006) compared with military personnel engaged in operational activities. Using the classification of body fat percentage below the average as the reference category, participants with aboveaverage body fat percentage demonstrated a 43.4% higher likelihood of developing BS (OR 1.434; 95%CI 1.0062.044; p = 0.046). Individuals with BS also had higher mean systolic blood pressure, and each 1 mmHg increase in systolic pressure was associated with an increased probability of BS (OR 1.013; 95%CI 1.001-1.025; p = 0.038). In addition, these participants had lower mean high-density lipoprotein cholesterol (HDL-c) levels; for each 1 mg/dL increase in HDL-c, the likelihood of burnout decreased, confirming its protective effect (OR 0. 988; 95%CI 0.979-0.998; p = 0.019). No statistically significant associations were identified for the remaining variables evaluated (Table 2).

**Table 2:** Association between demographic, occupational, nutritional, anthropometric, and clinical characteristics of participants with and without burnout

Characteristic	Burnout		OR	95%CI		OR
	With BS (n=293) n (%)	Without BS (n=309) n (%)		Lower	Upper	
Gender						
Female	73 (44.5)	91 (55.5)	1			
Male	220 (50.2)	218 (49.8)	1.258	0.877	1.804	0.212
Age group (years)						
21-30	105 (49.3)	108 (50.7)	1			
31-40	107 (47.3)	119 (52.7)	0.984	0.655	1.480	0.939
> 40	81 (49.7)	82 (50.3)	0.910	0.608	1.362	0.648
Rank						
Corporals/Privates	49 (54.4)	41 (45.6)	1			
Superior Officers	16 (64.0)	9 (36.0)	1.488	0.595	3.718	0.395
Captains/Lieutenants	83 (48.8)	87 (51.2)	0.798	0.478	1.333	0.389
Warrant Officers/Sergeants	145 (45.7)	172 (54.3)	0.705	0.441	1.129	0.146
Length of service (years)						
< 10	175 (48.5)	186 (51.5)	1			
> 10	118 (49.0)	123 (51.0)	1.020	0.736	1.413	0.907
Work category						
Operational	142 (43.6)	184 (56.4)	1			
Bureaucratic	151 (54.7)	125 (45.3)	1.565	1.134	2.161	0.006
Nutritional status						
Eutrophic	114 (50,4)	112 (49.6)	1			
Overweight/obesity	179 (47,6)	197 (52,4)	0.893	0.642	1.242	0.500
Classification of body fat (%)						
Better than average	87 (43.5)	113 (56.5)	1			
On average	36 (46.2)	42 (53.8)	1.113	0.658	1.883	0.689
Worse than average	170 (52.5)	154 (47.5)	1.434	1.006	2.044	0.046
Metabolic syndrome						
No	208 (46.6)	238 (53.4)	1			
Yes	85 (54.5)	71 (45.5)	1.370	0.950	1.975	0.092
Age (years)	34.1±9.4	34.3±9.2	0.998	0.981	1.016	0.837
BMI (kg/m^2^)	26.5±3.9	26.3±3.7	1.010	0.969	1.054	0.633
Weight (kg)	78.3±14.2	77.0 ±13.7	1.006	0.995	1.018	0.287
Body fat (%)	24.6±7.9	24.0±7.0	1.011	0.989	1.033	0.334
SBP (mmHg)	125.1±13.7	122.8±13.5	1.013	1.001	1.025	0.038
DBP (mmHg)	81.4±11.2	79.9±11.4	1.012	0.998	1.027	0.095
WC (cm)	86.0±10.9	84.8±9.8	1.012	0.996	1.027	0.145
Triglycerides (mg/dL)*	99 (72-144)	96 (70-139)	1.002	0.999	1.004	0.186
Glucose (mg/dL)	90.4±13.7	90.8±11.8	0.997	0.985	1.010	0.661
HDL-c (mg/dL)	50.1±16.1	53.2±16.8	0.988	0.979	0.998	0.019

Numerical variables are expressed as mean ± standard deviation, and categorical variables as absolute and relative frequencies. P-value: binary logistic regression. BMI = body mass index; BS = burnout syndrome; DBP = diastolic blood pressure; HDL-c = high-density lipoprotein cholesterol; OR = odds ratio; SBP = systolic blood pressure; WC = waist circumference.

* Median and interquartile range.

Table 3 illustrates how burnout levels across each MBI subscale varied among participant subgroups according to sociodemographic, metabolic, rank, and occupational characteristics. Professionals with high emotional exhaustion had a higher mean age (p = 0.001), belonged more frequently to the age group above 40 years, had over 10 years of service, and were predominantly Superior Officers or Captains/Lieutenants. In contrast, individuals with low emotional exhaustion were generally younger (21 to 30 years), had fewer than 10 years of service, and were more frequently classified in the Corporal and Private ranks (p = 0.008, p = 0.001, and p < 0.001, respectively). No significant associations were found between the analyzed variables and the depersonalization dimension. However, participants with low scores in the low personal accomplishment dimension (indicating greater accomplishment) were primarily Warrant Officers/Sergeants and those performing operational duties. Conversely, higher scores, reflecting lower personal accomplishment, were observed among Corporals and Privates (p = 0.007) and among those performing bureaucratic activities (p = 0.032). No other associations reached statistical significance.

**Table 3:** Participant’s level of burnout across the three MBI dimensions according to their characteristics

	Emotional exhaustion				Depersonalization				Low personal accomplishment			
	Low	Moderate	High	P	Low	Moderate	High	P	Low	Moderate	High	P
Gender				0.472^a^				0.154^a^				0.748^a^
Male	286 (65.3)	73 (16.7)	79 (18.0)		234 (53.4)	95 (21.7)	109 (24.9)		175 (40.0)	130 (29.7)	133 (30.4)	
Female	104 (63.4)	34 (20.7)	26 (15.9)		93 (56.7)	42 (25.6)	29 (17.7)		71 (43.3)	45 (27.4)	48 (29.3)	
Age (years)	33.4^c^±9.5	34.2^cd^±8.5	37.1^d^±8.7	0.001^b^	34.8±9.2	33.5±9.3	33.6±9.4	0.269^b^	34.9±9.3	34.3±9.1	33.2±9.4	0.168^b^
Age group (years)				0.008^a^				0.303^a^				0.197^a^
21-30	151 (70.9)	37 (17.4)	25 (11.7)		104 (48.8)	54 (25.4)	55 (25.8)		84 (39.4)	57 (26.8)	72 (33.8)	
31-40	140 (61.9)	47 (20.8)	39 17.3)		130 (57.5)	51 (22.6)	45 (19.9)		86 (38.1)	76 (33.6)	64 (28.3)	
> 40	99 (60.7)	23 (14.1)	41 (25.2)		93 (57.1)	32 (19.6)	38 (23.3)		76 (46.6)	42 (25.8)	45 (27.6)	
Length of service (years)				0.001^a^				0.199^a^				0.138^a^
<10	252 (69.8)	62 (17.2)	47 (13.0)		188 (52.1)	91 (25.2)	82 (22.7)		147 (40.7)	96 (26.6)	118 (32.7)	
>10	138 (57.3)	45 (18.7)	58 (24.1)		139 (57.7)	46 (19.1)	56 (23.2)		99 (41.1)	79 (32.8)	63 (34.8)	
Work category				0.181^a^				0.113^a^				0.032^a^
OPNL	214 (65.6)	63 (19.3)	49 (15.0)		185 (56.7)	77 (23.6)	64 (19.6)		147 (45.1)	94 (28.8)	85 (26.1)	
BRCT	176 (63.8)	44 (15.9)	56 (20.3)		142 (51.4)	60 (21.7)	74 (26.8)		99 (35.9)	81 (29.3)	96 (34.8)	
Rank				<0.001^c^				0.970^a^				0.007^a^
SO	12 (48.0)	2 (8.0)	11 (44.0)		14 (56.0)	4 (16.0)	7 (28.0)		9 (36.0)	7 (28.0)	9 (36.0)	
Cap./Lieut.	93 (54.7)	38 (22.4)	39 (22.9)		95 (55.9)	39 (22.9)	36 (21.2)		65 (38.2)	58 (34.1)	47 (27.6)	
WO/Sgt.	211 (66.6)	57 (18.0)	49 (15.5)		171 (53.9)	72 (22.7)	74 (23.3)		147 (46.4)	86 (27.1)	84 (26.5)	
Corp./Priv	74 (82.2)	10 (11.1)	6 (6.7)		47 (52.2)	22 (24.4)	21 (23.3)		25 (27.8)	24 (26.7)	41 (45.6)	
NTI status				0.198^a^				0.896^a^				0.759^a^
Eutrophic	149 (65.9)	45 (19.9)	32 (14.2)		120 (53.1)	53 (23.5)	53 (23.5)		90 (39.8)	64 (28.3)	72 (31.9)	
OO	241 (64.1)	62 (16.5)	73 (19.4)		207 (55.1)	84 (22.3)	85 (22.6)		156 (41.5)	111 (29.5)	109 (29.0)	
MetS				0.300^a^				0.162^a^				0.117^a^
No	296 (66.4)	78 (17.5)	72 (16.1)		235 (52.7)	110 (24.7)	101 (22.6)		193 (43.3)	126 (28.3)	127 (28.5)	
Yes	94 (60.3)	29 (18.6)	33 (21.2)		92 (28.1)	27 (17.3)	37 (23.7)		53 (34.0)	49 (31.4)	54 (34.6)	

Numerical variables are expressed as mean ± standard deviation, and categorical variables as absolute and relative frequencies.

BRCT = bureaucratic; Cap./Lieut. = Captains/Lieutenants; Corp./Priv. = Corporals and Privates; MBI = Maslach Burnout Inventory; MetS = metabolic syndrome;

NTI = nutritional; OO = overweight/obesity; OPNL = operational; SO = Superior Officers; WO/Sgt. = Warrant Officers/Sergeants; yr = year.

P-value: a = Pearson’s chi-squared test; b = ANOVA and post hoc Bonferroni; c = Fisher’s exact test. For Bonferroni’s post-hoc comparisons (superscript letters “c” and “d”): different letters denote statistically significant differences between means; identical letters indicate no significant difference.

**Figure 1:**
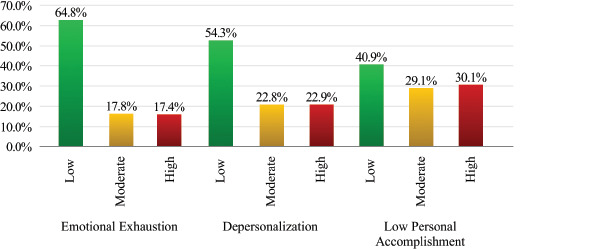
Classification of burnout levels according to Maslach Burnout Inventory subscales.

Figure 1 illustrates the distribution of burnout levels across the three MBI dimensions. The “low personal accomplishment” subscale demonstrated the most concerning pattern, with more than 30% of participants classified with high levels of burnout in this dimension.

## DISCUSSION

With the suspension of regular physical activities in military units, the Institutional Command issued a series of official guidelines on COVID-19 prevention for service members and their families. These recommendations advised the practice of social distancing, the use of masks, protective measures during military training, the need for adequate hand hygiene, and the disinfection of workspaces, among others. The rapid spread of the pandemic around the world prompted the cancellation of numerous collective activities, including ceremonies and sports competitions, requiring military personnel to maintain a state of permanent and even more vigilant readiness.

The results of this study revealed a high prevalence of BS among participants. In addition, the scores associated with the syndrome were elevated in half of the sample, with the exception of the “low personal accomplishment" dimension. This finding was particularly associated with individuals with longer career duration, high service rank, and those who performed bureaucratic activities.

After an extended period of suspended activities, many professionals were compelled to adapt by transforming their homes into improvised gyms and adopting creative strategies to maintain their fitness levels and avoid excessive detraining. Nevertheless, most personnel were negatively affected by the inability to exercise regularly, which made it impossible to apply the International Physical Activity Questionnaire in this study. The interruption of regular exercise has negative effects on cardiovascular conditioning, muscle function, and energy metabolism. Illness, injury, and travel are factors that can impair the vigor and health of military personnel, and the restrictions imposed by the COVID-19 pandemic added additional challenges.^13^,^14^

In this study, 48.7% of participants presented BS according to total MBI score, meaning that they showed high levels in at least one of the three dimensions. A survey conducted in Japan during the pandemic evaluated 312 health professionals with a mean age of 30.5 years and reported an overall BS prevalence of 31.4%.^15^ Another study carried out during the same period evaluated 100 medical residents in an emergency hospital in Romania and found a prevalence of 76%.^16^,^17^ In Iran, a cross-sectional study involving 615 participants in the first 2 months of the pandemic found that 53.0% experienced high levels of burnout. One of the most extensive studies examining BS in the pandemic context was conducted by Azoulay et al.,^18^ who evaluated 846 frontline physicians from 85 countries and reported a prevalence of 51.8%.^18^ Variations in the prevalence of BS across different regions and professional groups can be explained by occupational characteristics (type of activity performed, degree of autonomy, among others), as well as by different national responses to the pandemic, different containment measures, heterogeneous and asynchronous peaks of infection worldwide, and distinct levels of psychological pressure among different populations. Considering the lack of studies examining the overall prevalence of BS among armed forces’ members worldwide, comparisons among distinct emergency response sectors (eg, military personnel and health care professionals) may offer valuable insights into specific stress response patterns among different professional groups.

There is a predominance of research on BS involving health care and education professionals, whereas studies focusing on military personnel, particularly within the armed forces, are scarce. The only study to date evaluating the overall prevalence of BS among Brazilian Army professionals outside the health sector was conducted in 2016 and reported a surprising prevalence of 89% among 119 combatants in an infantry battalion.^2^ However, this percentage should be interpreted with caution, given that the investigation was limited to a single battalion and involved a relatively young sample. Selection bias may have influenced the findings, as younger individuals, in addition to having had little time to adapt to military routines, may have been subjected to highly specific working conditions, depending on the internal regulations of that particular military unit.

In the present study, professionals performing bureaucratic activities were 56.5% more likely to develop BS compared with those performing operational tasks. Similar observations have been reported in other fields: a study involving U.S.

Graduate Medical Education (GME) faculty members found that heavy bureaucratic demands were among the most common triggers for BS. According to the authors, such conditions could contribute to insomnia, depression, and security-related incidents in the military health system.^19^ Although the growing bureaucratization of contemporary institutions is intended to improve organizational efficiency, particularly where administrative support is insufficient and productivity metrics are emphasized, these same bureaucratic pressures may become a significant source of emotional exhaustion, depersonalization, and low personal accomplishment among staff.^20^

Using the below-average body fat percentage category as the reference, participants with aboveaverage body fat presented a 43.4% higher likelihood of developing BS. Teisala et al.^21^ reported an association between increased stress levels and higher body fat percentage.^21^ Tension at work may contribute to increased adiposity, as stress often leads to unhealthy lifestyle choices — such as physical inactivity and inadequate diet — that promote weight gain and high levels of body fat. However, this relationship is bidirectional, given that obesity can impair work capacity and increase the risk of experiencing stress, a phenomenon described as the reverse causality hypothesis.^22^

According to the MBI subscales, 17.4% of participants had high levels of emotional exhaustion. A higher proportion (22.9%) showed elevated depersonalization scores. More concerning was the finding that 30.1% scored in the high range for low personal accomplishment. Lazaro-Perez et al.^23^ evaluated members of the Spanish Security Forces during the pandemic and reported prevalence rates of 53.8%, 58.4%, and 27.0% for emotional exhaustion, depersonalization and low personal accomplishment, respectively. Based on these subscale distributions, Brazilian military personnel exhibited comparatively lower levels of burnout than their Spanish counterparts. This difference may be associated with the more intense stressors reported in the Iberian Peninsula during that period, including increased security interventions due to noncompliance with public health regulations, in addition to various environmental disasters such as fires and floods,^23^ all of which are undoubtedly capable of exacerbating burnout.

Another study conducted in the Islamic Republic of Iran, involving 1,209 military personnel with a mean age of 36.5 years, reported high burnout levels across all MBI dimensions: emotional exhaustion (54.6%), depersonalization (56.6%), and low personal accomplishment (57.9%).^24^ Again, these rates were substantially higher than those observed among Brazilian personnel in the present study. This discrepancy may be explained by the persistent state of tension experienced by members of the Iranian Armed Forces, given the hostile geopolitical context involving the United States, its Gulf allies, and Israel, with an ongoing and concrete risk of military confrontation. These findings underscore that, regardless of the consequences of the pandemic, it is essential to understand the psychosocial demands of Army professionals to promote optimal occupational health conditions.

Military personnel with high emotional exhaustion were predominantly those older than 40 years, with more than 10 years of service, and occupying higher ranks. Career progression, combined with extended length of service, appears to increase work demands and the burden of responsibilities. When coping alternatives for managing stress are limited, these factors may predispose armed forces personnel to heightened tension and emotional exhaustion.^25^ Our results are in accordance with the observations of Ojvodic & Dedic,^26^ who attribute stress in the military profession to a combination of routine occupational stressors. Unpredictable working hours, field training activities, the constant possibility of reassignment to another unit or city, and the expectation of maintaining exemplary conduct as a model for subordinates are all aspects that tend to be more prominent in the daily lives of more experienced service members.^26^

The association between the low personal accomplishment subscale and the bureaucratic category of work suggests that administrative duties may contribute to professional dissatisfaction. Supporting this observation, a survey conducted with professionals at a hospital in Montes Claros, Minas Gerais, Brazil, reported high levels of occupational stress among individuals assigned to bureaucratic functions. According to the authors, administrative activities and interpersonal relationships are key factors that trigger stressful situations in the workplace.^27^ In military units, administrative activities are currently subject to numerous control mechanisms, high stress levels arising from excessive demands, and even legal uncertainties surrounding the execution of certain duties. These circumstances may be responsible for triggering feelings of emotional and psychological vulnerability. Other comparative analyses were not pursued, as the use of different assessment scales and distinct cut-off criteria introduces inherent heterogeneity.

Despite the lack of studies examining BS during periods of population-wide restrictive measures, the present study contributes to the growing body of evidence on the potential psychological burden associated with the COVID-19 pandemic. The crosssectional design represents a limitation, as it restricts causal inference and precludes comparisons with pre-pandemic data. In addition, although the sample size was considerable, the study was restricted to a single military region in Brazil. Future studies should consider a longitudinal design.

Military personnel in Brazil are rarely included in epidemiological studies, and there is a lack of research addressing the specific characteristics of the profession and its association with the development of illness. Considering their essential role in the protection and safety of citizens — such as installing field hospitals, disinfecting public facilities, transporting medications and equipment, performing numerous other activities during the pandemic under continuous exposure to the virus — this population warrants special attention and recognition. In this regard, further studies involving different regions of the country, as well as other branches of the Armed Forces, are recommended to support broader discussions on the health of these professionals who play a vital role in society.

## CONCLUSIONS

The overall prevalence of BS observed in this study was high, affecting almost half of the sample. The findings indicate that performing bureaucratic functions and presenting above-average body fat percentage were both associated with the development of BS. Additionally, individuals aged over 40 years, those holding higher military ranks, and those with more than 10 years of service showed higher levels of emotional exhaustion.
